# Chemotherapy-Exacerbated Breast Cancer Metastasis: A Paradox Explainable by Dysregulated Adaptive-Response

**DOI:** 10.3390/ijms19113333

**Published:** 2018-10-26

**Authors:** Justin D. Middleton, Daniel G. Stover, Tsonwin Hai

**Affiliations:** 1 Molecular, Cellular, and Developmental Biology Program, Department of Biological Chemistry and Pharmacology, The Ohio State University, Columbus, OH 43210, USA; Middleton.124@buckeyemail.osu.edu; 2 Department of Internal Medicine, The Ohio State University, Columbus, OH 43210, USA; Daniel.Stover@osumc.edu; 3 Department of Biological Chemistry and Pharmacology, Molecular, Cellular, and Developmental Biology Program, The Ohio State University, Columbus, OH 43210, USA

**Keywords:** chemotherapy, breast cancer metastasis, stress response, adaptive-response network, ATF3, seed and soil theory, cancer-host interaction, tumor microenvironment, immune modulation, tumor immune environment

## Abstract

An emerging picture in cancer biology is that, paradoxically, chemotherapy can actively induce changes that favor cancer progression. These pro-cancer changes can be either inside (intrinsic) or outside (extrinsic) the cancer cells. In this review, we will discuss the extrinsic pro-cancer effect of chemotherapy; that is, the effect of chemotherapy on the non-cancer host cells to promote cancer progression. We will focus on metastasis, and will first discuss recent data from mouse models of breast cancer. Despite reducing the size of primary tumors, chemotherapy changes the tumor microenvironment, resulting in an increased escape of cancer cells into the blood stream. Furthermore, chemotherapry changes the tissue microenvironment at the distant sites, making it more hospitable to cancer cells upon their arrival. We will then discuss the idea and evidence that these devastating pro-metastatic effects of chemotherapy can be explained in the context of adaptive-response. At the end, we will discuss the potential relevance of these mouse data to human breast cancer and their implication on chemotherapy in the clinic.

## 1. The Double-Edged Sword of Chemotherapy-Findings from Mouse Models

### 1.1. The Paradox of Chemotherapy

Although tumors can be reduced to undetectable level by modern chemotherapy, in many cases they recur at the original or distant sites. Traditionally, this was thought to be a manifestation of “survival of the fittest”: The chemotherapeutic drugs exert selection pressure that allowed resistant cancer cells to survive, grow, and eventually thrive. However, emerging pictures from cancer research in the last decade showed that, paradoxically, chemotherapy can actively induce changes that favor cancer progression. These pro-cancer changes can be either inside (intrinsic) or outside (extrinsic) the cancer cells. For intrinsic changes, chemotherapeutic drugs have been shown to up-regulate the expression of anti-apoptotic genes [1], and to increase the ability of cancer cells to migrate/invade [2,3]. For extrinsic changes, chemotherapeutic drugs have been shown to change the non-cancer cells within the host—the organism that carries the cancer cells (some reviews, ([4,5,6,7,8]). Note that the issue at hand here is the pro-cancer effect of chemotherapy, rather than the well-recognized side effect of chemotherapy, such as nausea and hair loss.

Although the field is relatively new, it has made significant advancement by leveraging the extensive knowledge on cancer-host interaction (a few reviews, such as References [9,10,11,12,13]). Intensive research in the past few decades has demonstrated that cancers are not simply autonomous masses of cells. They secrete soluble factors and exosomes (extra-cellular microvesicles) to elicit systemic responses from the host. The host in turn sends soluble factors and bone marrow-derived precursor cells (hematopoietic and mesenchymal) to the tumors and the future metastatic sites to affect cancer progression, forming a loop of cancer-host interaction (above reviews). Relevant to our discussion here is the myeloid-lineage of cells, particularly the macrophages, which play a key role for the host to enhance cancer progression. The ability of these cells to promote cancer progression seems counter-intuitive, since the main function of macrophages is to fight against infection and eliminate damaged cells. A widely accepted explanation is that macrophages in the tumor, called tumor-associated macrophages (TAMs), are educated by cancer cells over time, and are converted from anti-cancer to pro-cancer, at least in part, by changing their gene expression (some reviews, References [14,15,16]). For the complexities and nuances of myeloid cells in cancer progression, see aforementioned reviews. It was against this backdrop of cancer-host interaction that various studies showed the pro-cancer effect of chemotherapy.

### 1.2. The Pro-Cancer Effect of Chemotherapy—Chemo-Resistance versus Chemo-Exacerbation

#### 1.2.1. Chemo-Resistance: Chemotherapy Counteracts Its Own Efficacy

Less than a decade ago, several papers published within two years of each other demonstrated that chemotherapeutic agents (such as paclitaxel, doxorubicin, and gemcitabine) increased the abundance of TAMs in primary tumors in breast and other cancer models [17,18,19,20]. Since TAMs promote cancer progression, it is surprising and alarming that chemotherapy—a treatment to fight against cancer—can actually increase the abundance of TAMs. Functionally, depletion or inhibition of TAMs by inhibitors or genetic manipulation improved the efficacy of chemotherapy—as evidenced by the further reduced tumor size, lower metastatic burden, but higher survival rate ([17,18,19,20]; for a few reviews, see References [5,6,7]). Thus, by increasing the recruitment of TAM (a non-cancer host cell), chemotherapy can paradoxically elicit pro-cancer effect and counteract its own efficacy. In this review, we refer to this as a chemotherapy-induced chemo-resistance, in order to distinguish it from chemotherapy-exacerbated metastasis discussed below (Section 1.2.2). Note that the studies above uncovered a hidden pro-cancer effect of chemotherapy that can be dampened by inhibiting TAMs. However, they did not show any increase in metastasis by chemotherapy, a phenomenon shown in the studies discussed below (Section 1.2.2). This discrepancy can be explained by several possibilities. First, most of the papers above compared chemotherapy alone to chemotherapy with the depletion or inhibition of TAMs, but did not show the comparison between chemotherapy and control (vehicle) therapy. Thus, it is not possible to discern whether chemotherapy exacerbated metastasis. Second, although Shree et al. showed no difference between chemotherapy and control treatment [20], many factors can influence the results, such as cancer models, treatment regimen, and the time point for analyses.

#### 1.2.2. Chemo-Exacerbation: Chemotherapy Exacerbates Metastasis

Metastasis is a multi-step process composed of cancer cell escape from the primary tumor, survival in circulation, and colony formation at the distant site. Since metastasis is the major cause of cancer death, it is important to address the effect of chemotherapy on metastasis. A recent review [21] extensively discussed the metastatic cascade and the potential ways that chemotherapy could affect each step of the cascade. In this section, we will discuss a few studies demonstrating chemotherapy-enhanced metastasis using mouse models [3,22,23,24,25,26,27,28]. Two reports addressed the effect of chemotherapy on the intrinsic properties of cancer cells. Volk-Draper et al. reported that paclitaxel enhanced metastasis by activating the Toll-like Receptor 4 (TLR4) signaling in cancer cells, which in turn increased systemic inflammation and myeloid cell outgrowth [22]. Ren et al. reported that high dose paclitaxel induced cancer cell invasion in vitro and increased metastasis in vivo in a manner dependent on the miR-21/CDK5 axis [3]. Below, we discuss the exacerbation of metastasis by chemotherapy via the modulation of non-cancer cells (the focus of this review).

Insight from the experimental metastasis model: Using the experimental metastasis model, several groups demonstrated that chemotherapy creates a tissue environment at the distant site that is favorable for cancer cells to colonize upon their arrival [23,24,25,27]. In the experimental metastasis model, cancer cells are delivered into the blood stream by intravenous or intracardiac injection. Because the mice do not have primary tumors, this model does not examine the ability of cancer cells to escape from the primary tumors; instead, it only examines the ability of cancer cells in circulation to colonize the target tissue (such as lung, bone or liver, depending on the cancer cells). To test whether chemotherapy affects cancer colonization at the distant site, researchers pre-treated the mice with chemotherapeutic agents and then injected cancer cells after the drugs had been eliminated from the mice. Because of this delay in cancer cell injection, any effect of chemotherapy on cancer burden would be due to its effect on the non-cancer host cells, which in turn affects the ability of cancer cells to colonize the target tissues. All drugs tested in this model (paclitaxel, gemcitabine, cisplatin, and cyclophosphamide) enhanced cancer burden at the target tissues [23,24,25,27], indicating that chemotherapy creates a favorable tissue environment for cancer cells to colonize. In some studies [23,27], cancer burden was examined shortly (within three days) after injection in order to analyze seeding—the ability of circulating cancer cells to extravasate and survive in the tissue parenchyma, but before major outgrowth. These studies indicated that chemotherapy also exacerbated seeding. Since chemotherapy-exacerbated tissue colonization by cancer cells is observed in several cancer cell models (breast, prostate, colon, lung and melanoma) using multiple drugs (see above), it is likely a broadly applicable phenomenon.

**Figure 1:**
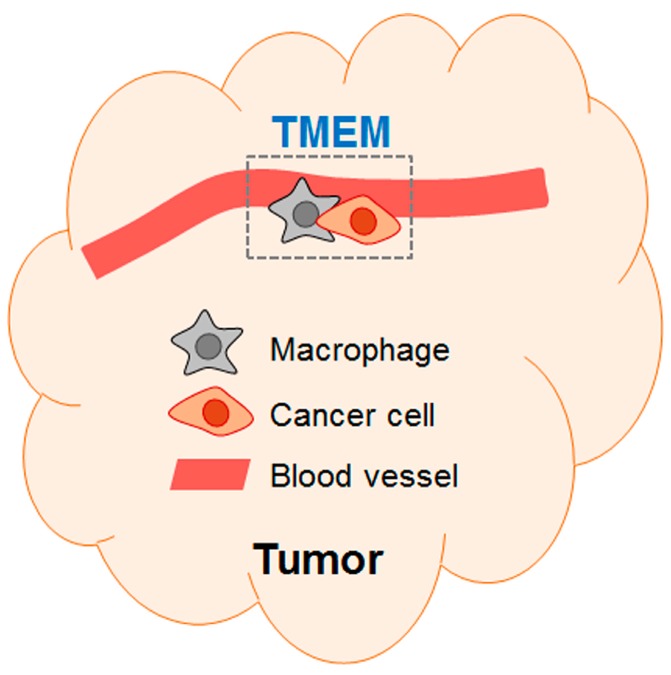
A schematic of TMEM (tumor microenvironment metastasis). The schematic shows a TMEM composed of a macrophage and a cancer cell at peri-vascular location (first named by Robinson et al. [29]).

Insight from the spontaneous metastasis model: Researchers also studied the effect of chemotherapy on metastasis using the spontaneous metastasis model, where cancer cells are injected at the orthotopic site to give rise to primary tumors, followed by chemotherapy and analyses of cancer (see References [26,27,28]). These studies used breast cancer models and showed that, despite reducing the tumor size, paclitaxel increased metastasis. Alishekevitz et al. showed that paclitaxel increased lymphatic density in the tumors with an accompany of increased VEGFR3^+^ macrophages. Their data from antibody blocking experiments indicated that the VEGF-C/VEGFR3 axis is important for paclitaxel to increase cancer cell dissemination via the lymphatic system [26]. Interestingly, paclitaxel can also increase the dissemination of cancer cells via the vascular system. Two contemporaneous papers (Chang et al. and Karagiannis et al. [27,28]) showed that paclitaxel increased the abundance of a micro-anatomical structure called tumor microenvironment of metastasis (TMEM). This structure is composed of a macrophage and a cancer cell in close proximity at the peri-vascular location [29] as diagramed in Figure 1. Importantly, intravital imaging showed that this is the site where cancer cells enter the blood stream [30]. Consistent with the increase in TMEM, both studies [27,28] showed increased circulating cancer cells and enhanced metastasis by paclitaxel. Therefore, despite its apparent benefit of reducing tumor size, paclitaxel exacerbated metastasis. We note that, in the above three studies using the spontaneous metastasis models [22,26,27,28], chemotherapy was administered while primary tumors were still present. Thus, they mimic the neoadjuvant (pre-operative) chemotherapy, not the adjuvant (post-operative) chemotherapy. This has implication on how to interpret these data in clinical consideration (see below, Section 4.1).

Chang et al. also analyzed the lung, the metastatic site of their models [27]. Among other things, paclitaxel increased the abundance of inflammatory monocyte (iM., which is known to differentiate into metastasis-associated macrophages [31]) and suppressed the anti-cancer immune microenvironment. Thus, paclitaxel enabled more cancer cells (the seeds) to escape from primary tumors, and made the lung microenvironment (the soil) more hospitable to cancer cells, explaining the paradoxical ability of chemotherapy to exacerbate metastasis in the context of the “seed and soil” theory [32].

### 1.3. Pre-Metastatic versus Metastatic Niche

It is well known that primary tumors secrete soluble factors and exosomes to change the microenvironment at the distant tissues, making them conducive to cancer survival and outgrowth before the arrival of cancer cells—an environment called pre-metastatic niche (see Reference [33]). Upon arrival, cancer cells can further modulate the distant tissues to make them more hospitable—an environment called metastatic niche. In the experimental metastasis model (see above Section 1.2.2), chemotherapy pre-treatment increased the ability of cancer cells to colonize the target tissues, suggesting that chemotherapy created a pre-metastatic niche for cancer cells. However, strictly speaking, the data only indicate that chemotherapy facilitated a more favorable tissue environment—without distinguishing pre-metastatic versus metastatic niche. This is because the assays in those studies were carried out after the injection of cancer cells. To address the issue of pre-metastatic niche, one needs to analyze the target tissues for molecular and/or cellular changes that contribute to cancer survival and outgrowth—without cancer cell injection. A tantalizing result from Daenen et al. suggests that chemotherapy may indeed create a pre-metastatic niche in the absence of any signals from cancer cells. They found that, four days after cisplatin injection with no cancer cells, the expression of VEGFR-1 is up-regulated in the activated (VCAM1^+^) endothelial cells [23]. However, they did not show whether this up-regulation functionally contributed to cisplatin-exacerbated cancer burden. Clearly, it is important to address whether chemotherapy can induce pre-metastatic niche formation. If yes, what are the underlying mechanisms? Do they share any common elements with the pre-metastatic induction by signals from primary tumors? 

### 1.4. The Culprit in the Host—the Key Non-Cancer Cells Contributing to the Pro-Cancer Effect of Chemotherapy

Since chemotherapy is given systemically, it can affect all host cells. Thus far, myeloid cells and endothelial cells have been identified to play important roles for chemotherapy to induce pro-cancer activities.

#### 1.4.1. Myeloid Cells

The myeloid lineage contains subsets of cells, such as macrophages and monocytes (macrophage precursor), neutrophils, and others. The nomenclature is complicated by the fact that each subset of myeloid cell is heterogeneous in nature, and that people used overlapping, but not exactly the same markers or names. As such, cells with the same names may not be identical, but only share some markers, and cells with different names may be similar or the same. In this review, we will use the simple definition CD11b^+^, F4/80^+^ for macrophage. We will focus on the roles of macrophage (or monocyte) in the pro-cancer effect of chemotherapy, because the majority of the current literature is for them.

As indicated in Section 1.1, TAMs are well documented to promote cancer progression. Therefore, it is not surprising that macrophage is a key conduit for chemotherapy to bring about its pro-cancer effects. In principle, chemotherapy can modulate macrophages by increasing their abundance and/or changing their properties. This has been demonstrated to be the case. Table 1 summarizes a few reports, showing the increase of macrophage abundance in the tissue or sub-tissue localization, and the changes of macrophage properties, such as inflammasome activation and increased bioactivity. The mechanisms of macrophage action can be classified into three categories: (a) Macrophages alter cancer cell behavior or activity to make them more successful (such as, better survival, more invasive, more tumor initiation); (b) macrophages suppress cytotoxic T cells, thus protecting cancer cells from immune attack; and (c) macrophages alter the blood or lymphatic vessels to help cancer cells. All three categories are well-known mechanisms by which macrophages promote cancer progression. The new information here is the ability of chemotherapy to modulate macrophages. As such, only reports with this information are included in the Table. Reports without this information are not included, even if they showed a link between macrophages and chemotherapy—in the direction of macrophage affecting chemotherapy. As an example, Jinushi et al. [34] showed that TAMs secrete milk-fat globule epidermal growth factor VIII (MFG-E8), which confers the ability of tumor initiation cells (TICs) to promote tumorigenicity and chemoresistance. Although the report demonstrated the importance of MFG-E8 to influence TICs in terms of chemo-resistance, it did not show whether chemotherapy affects TAMs. Therefore, it is not included in the Table. We note that the Table is not meant to be comprehensive, but to provide some examples of macrophage modulations by chemotherapy. The ultimate consequence is that chemotherapy, by modulating macrophages, paradoxically promotes cancer progression, either as a hidden feature that counteracts its own therapeutic efficacy or as an apparent exacerbation of metastasis (see chemo-resistance versus chemo-exacerbation discussed in Section 1.2).

All the above literature is for macrophages in the primary tumors. The literature on macrophages at the distant site—in the context of chemotherapy—is scarce. Chang et al. showed that paclitaxel increased the concentration of CCL2, a recruitment factor for myeloid cells, in the lung. This is accompanied by an increased abundance of iMs and decreased anti-cancer immune microenvironment. Furthermore, their data support the idea that the subsets of myeloid cells that are functionally important at the metastatic sites are different from those at the primary tumors, an idea proposed previously [6,7]. Clearly, more investigations are required to elucidate how chemotherapy, by affecting the myeloid cells at the distant sites, may promote a hospitable environment for cancer cells.

#### 1.4.2. Endothelial Cells

Although not extensive, the literature has shown several ways that chemotherapy can affect endothelial cells to promote cancer. (a) Chemotherapy can induce endothelial cells to secrete IL6 and tissue inhibitor of metallopeptidase (TIMP1), creating an environment that increases cancer cell survival [39]. (b) Chemotherapy promotes endothelial progenitor cells to mobilize to the tumor, thus enhancing cancer progression [40,41]. (c) In vitro analyses indicated that chemotherapy can increase the adhesiveness of endothelial cells, providing a potential explanation for the ability of chemotherapy to increase lung colonization by cancer cells in the experimental metastasis model [23].

**Figure 2:**
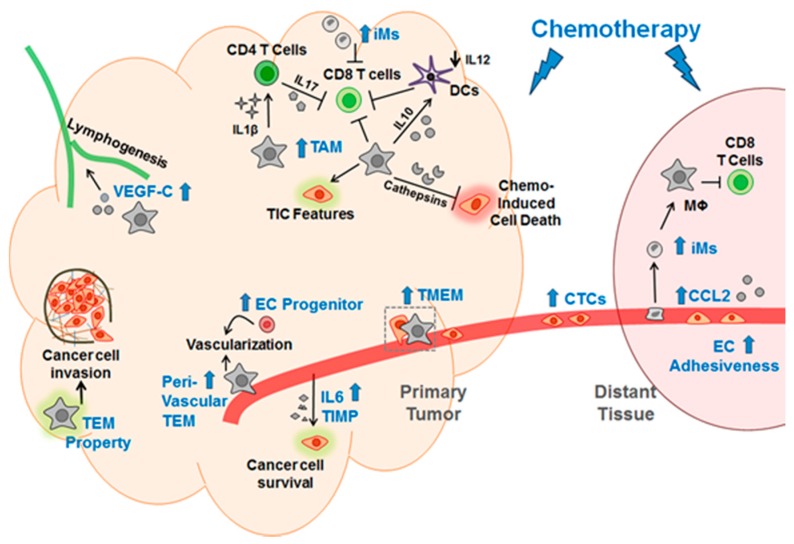
A schematic for the mechanisms by which chemotherapy elicits its pro-cancer effect via modulations of macrophages and endothelial cells. Blue text indicates the changes induced by chemotherapy; blue arrow denotes increase induced by chemotherapy; black arrow indicates promoting the events; black down arrow indicates decrease. Mϕ, macrophage; TEM, Tie2-expressing macrophage; TMEM, tumor microenvironment metastasis; EC, endothelial cells; iM, inflammatory monocyte; DCs, dendritic cells; CTC, circulating cancer cells; TIC, tumor initiation cell; TIMP, tissue inhibitor of metallopeptidase; the green and pink shades denote that the corresponding cells are altered.

Taken together, chemotherapy has been shown to elicit its pro-cancer effect by modulating myeloid cells and endothelial cells. Figure 2 shows a schematic summary of Section 1.2, Section 1.3 and Section 1.4.

## 2. Explaining the Pro-Cancer Effect of Chemotherapy from the Perspective of Adaptive-Response

Chang et al. further demonstrated that the pro-cancer effects of paclitaxel (such as increasing TMEM, iM, and metastasis) were dependent on a stress-inducible gene *Atf3* in the non-cancer host cells [27]. Using a spontaneous metastasis model (fat pad injection of cancer cells), they showed that paclitaxel exacerbated the ability of breast cancer cells to metastasize in the wild type (WT) mice, but not much in the knockout (KO) mice deficient in *Atf3*. Since the same breast cancer cells were injected into the mice and the only difference was the host, it means that paclitaxel exerted its pro-metastatic effect by affecting the host cells. The genotype difference between the mice indicates that the processes regulated by activating transcription factor 3 (ATF3) in the host cells are key mediators for paclitaxel to exacerbate metastasis. ATF3 is a transcription factor and the expression of its corresponding gene is induced by a variety of stress signals, including DNA damage, ischemia-reperfusion, seizure, wounding, endoplasmic reticulum stress, nutrient deprivation, cytokines, and chemotherapeutic agents (see References [42,43]). One striking feature of Atf3 induction is that it is neither stimulus- nor cell type-specific. The broad spectrum of signals to induce *Atf3* in all cell types examined thus far, in combination with other clues, prompted the idea that *Atf3* is a hub of the adaptive-response network responding to stress signals that disturb the cellular homeostasis [43]. Although many genes have been identified as target genes of ATF3, one common function of ATF3 appears to modulate immune response [43]. Thus, *Atf3* links stress signals to immune response. Since stress conditions and dysregulation of immune function can lead to the pathogenesis of many diseases, *Atf3* is likely a linchpin to the understanding of various diseases.

The *Atf3* gene is located on human chromosome at 1q32.3 within the 1q amplicon, which is the most frequently amplified region in human breast tumor: ~53% [44]. This implied that *Atf3* might play a role in human breast cancer. However, data supporting this notion did not emerge until a decade ago. *Atf3* gene expression was shown to be increased in human breast tumors [45,46]. Subsequent work collectively provided several lines of evidence supporting a role of *Atf3* in breast cancer. (a) *Atf3* functions as an oncogene in malignant breast cancer cells, such as increasing the TIC features of cancer cells and promoting tumor formation [45,46,47]. (b) *Atf3* amplifies the TGFβ signaling pathway [45,47] and activates the Wnt/β-catenin pathways [48]. (c) Although functionally important in the breast cancer cells (a and b), *Atf3* expression in the cancer cells does not correlate with worse outcome in breast cancer patients. Rather, it is the expression of *Atf3* in the non-cancer stromal cells—specifically the mononuclear immune cells—that correlated with worse outcome [49]. One explanation for this surprising result is that *Atf3* is induced in the mammary epithelial cells during their transformation into cancerous and malignant cells. These cancer cells then induce changes in the stroma. When the stroma starts to express *Atf3*, it reflects a reactive tumor microenvironment and dysregulated immune function. Since immune dysfunction plays a critical role in promoting metastasis, this may explain the value of stromal, but not cancer, *Atf3* to predict outcome. (d) Studies using breast cancer models comparing WT and KO mice indicated that *Atf3* in the non-cancer cells promotes metastasis. Analyses of conditional KO mice indicated that myeloid cell is a key cell type for this *Atf3* action [49]. (e) As a transcription factor, ATF3 modulates various target genes, and an ATF3 downstream gene-signature was identified to associate with worse outcome in a cohort of human breast cancer patients [49]. These findings, in conjunction with the stress-inducible nature of *Atf3*, formed the background for the studies by Chang et al. [27], which showed a necessary role of *Atf3* in the host cells to mediate chemotherapy-exacerbated metastasis (above).

**Figure 3:**
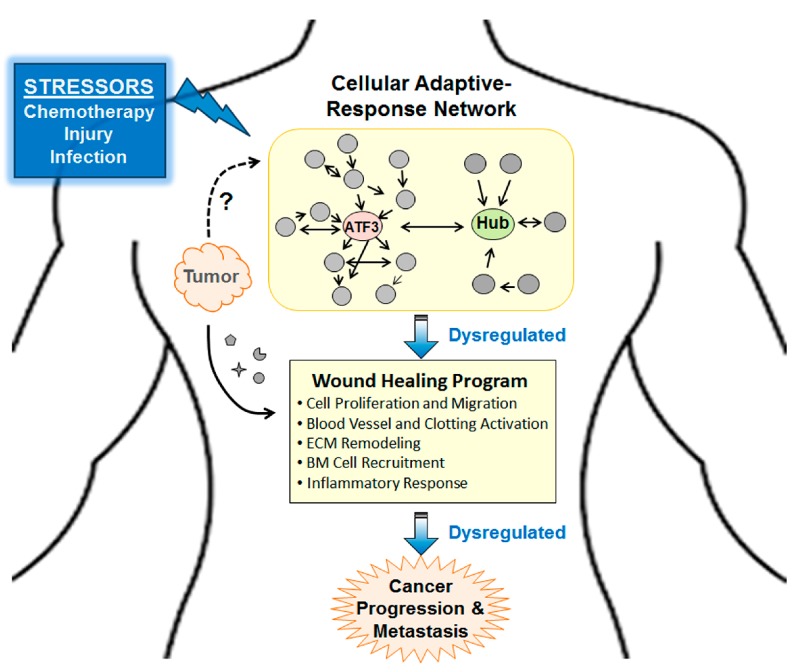
A schematic for the “dysregulated adaptive-response hypothesis.” Briefly, dysregulation of cellular adaptive-response network plays a central role for seemingly different stressors, such as chemotherapy, tissue injuries, and tumor signals to enhance cancer progression and metastasis. The “wound healing program” denotes a generic program entailing the indicated processes (in bullet points). However, detailed molecules or genes involved may vary in different context. ECM, extracellular matrix; BM, bone marrow.

As described above, *Atf3* is induced by many stress signals, not just chemotherapeutic agents. Thus, *Atf3* may also play a role in the ability of non-chemotherapy related stressors to facilitate metastasis, such as infection, traumatic injury, and even incisional surgery [33,50,51,52,53,54]. The surgery-enhanced metastasis has been referred to as “therapy at a cost” [54]. In this context, the following ideas are of particular interest: (a) Tumors have been referred to as wounds that never heal [55]. (b) The wound healing program is hijacked by tumor to help cancer cell survive and progress [4,56]. We note that wound healing and cancer progression/metastasis—to the first approximation—entail the same biological processes: (i) Stimulate cell proliferation and migration, (ii) activate blood vessels and clotting system, (iii) remodel extracellular matrix (ECM), (iv) recruit hematopoietic and mesenchymal precursor cells from bone marrow, and (v) modulate inflammatory response (for wound healing, see References [57,58]; for cancer progression/metastasis, see References [13,33,59,60,61]). We propose a “dysregulated adaptive-response hypothesis” as follow. Both tumors and injured (or infected) cells send out signals that disturb homeostasis, signals in the forms of soluble factors (such as cytokines, proteases, S100s), exosomes, and others. They would activate the cellular adaptive-response network. When this network is dysregulated over chronic conditions, pathological changes ensue. Presumably, *Atf3*, as a hub in the adaptive-response network, will be a linchpin for seemingly different stressors, such as tumor signals, chemotherapy, and traumatic injury, to enhance cancer progression and metastasis. Figure 3 shows a schematic of this hypothesis. Clearly, much more work is required to test this hypothesis. We note that various stress pathways, such as the DNA damage response and the integrated stress response pathway, have been shown to affect how cancer cells respond to chemotherapeutic agents (see References [62,63,64]). However, these pathways are predominantly examined in the context of stress response within cancer cells. Since the focus of this review is non-cancer cells, we do not discuss them here. 

## 3. The Relevance of the above Findings to Human Breast Cancer

Although mouse models are widely used in pre-clinical studies, data from them may not be extrapolatable to human. One way to address this issue is to test whether the molecular or cellular features identified in the mouse models are reflected in patient samples. Many of the above reports (discussed in Section 1) contain data from patient samples to support the relevance of their findings. As an example, analysis of publicly available datasets from human breast tumors showed that *Atf3* expression was higher in the breast tumor stroma from patients with chemotherapy than those without [27]. Furthermore, analyses of microarray datasets derived from the metastatic organs of human breast cancer patients showed that *Atf3* expression correlated with lower cytotoxic immune cell markers, consistent with the ATF3-associated immune suppression in mouse models. As another example, Karagiannis et al. analyzed 20 breast cancer tumors before and after neoadjuvant chemotherapy, and found increased TMEM abundance by chemotherapy [28]. Since higher TMEM abundance correlated with worse outcome [29], these results suggest that neoadjuvant chemotherapy may have undesirable long-term consequences. Taken together, data from preclinical research using mouse models support the notion that chemotherapy can enhance metastasis and that this paradoxical effect of chemotherapy is likely to have human relevance.

## 4. Should Findings from Mouse Models Affect Clinical Practices?

Chemotherapy is a longstanding treatment for cancer patients and has been shown to cure some blood cancers, such as childhood leukemia and adult Hodgkin’s lymphoma (see Reference [65]). Thus, it would not be prudent to change clinical practices without further investigation. However, the data discussed above indicate that it may be possible to improve the efficacy of chemotherapy by inhibiting its paradoxical pro-cancer effect. Below, we discuss neoadjuvant and adjuvant chemotherapy separately.

### 4.1. Neoadjuvant (Pre-Operative) Chemotherapy

In neoadjuvant setting, chemotherapy is administered before tumor removal. The advantages of this treatment modality include reducing tumor size for operation, increasing breast conservation, providing prognostic information based on tumor’s responsiveness to the treatment, and offering optimal setting for research [66]. Importantly, patients have been shown to benefit from neoadjuvant chemotherapy in clinical trials (see Reference [67]). This may appear contradictory to the findings discussed above that, in mouse models mimicking neoadjuvant chemotherapy, the treatment enhanced metastasis [22,26,27,28]. One potential explanation is that none of those mouse studies removed the tumors before end-point assays. Therefore, the conditions are not the same as those in the clinics. What those studies suggest is that exposing patients to chemotherapy while their tumors are still present could change the biological properties of the tumors and lead to undesirable consequences. These include increased lymphogenesis [26] and higher density of TMEM [27,28], both of which can allow more cancer cells to escape from the primary tumors. Thus, the benefits of neoadjuvant therapy need to be weighed against the potential undesirable effect. We surmise that the treatment can be improved by personalized medicine based on individual patients’ condition, such as tumor immune-microenvironment. As an example, DeNardo et al. showed that leukocyte complexity can predict patients’ response to neoadjuvant chemotherapy [17]. Tumors with low macrophage, but high cytotoxic T cells (CD68^low^/CD8^high^) responded better to therapy than those with high macrophage, but low CD8-T cells (CD68^high^/CD8^low^): 27% pathologic complete response versus 7%. Considering the potential detrimental effect of neoadjuvant chemotherapy, careful analysis of individual’s conditions and further investigation, including clinical trials are warranted.

### 4.2. Adjuvant (Post-Operative) Chemotherapy

Adjuvant chemotherapy removes tumors first before treating the patients with chemotherapy, and is considered the standard of care, except in the cases of inoperable disease [66]. When patients with adjuvant chemotherapy were compared to those with surgery only, adjuvant chemotherapy has been shown to reduce recurrence and increase overall survival [68,69,70]. However, as discussed in the section on metastasis, chemotherapy modifies the tissue environment—the soil—at the distant site and makes it more hospitable to cancer cells [27]. Thus, if any cancer cells that disseminated before or during tumor removal can survive chemotherapy, they will have a chance to recur and flourish. This may explain that, in sub-populations of patients, the disease comes back with a vengeance after chemotherapy. Traditionally, this was viewed as the result of “survival of the fittest,” where cancer cells with the most aggressive mutations managed to emerge and succeed. With the insight that chemotherapy can elicit a tissue environment favorable to cancer cells, we now have a new avenue to potentially improve chemotherapy. By elucidating the mechanisms behind this effect, we may be able to dampen the undesirable ability of chemotherapy to modify the soil, thus increasing the therapeutic efficacy of adjuvant chemotherapy.

## 5. Conclusions

In summary, recent studies from mouse models demonstrated that chemotherapy can paradoxically enhance cancer progression. This review focuses on the impact of chemotherapy on the non-cancer host cells. Chemotherapy was shown to counteract its own efficacy by modulating TAMs, a phenomenon we referred to as chemotherapy-induced chemo-resistance. Chemotherapy was also shown to increase breast cancer metastasis by increasing the escape of cancer cells (seeds) from the primary tumors and by creating a more favorable tissue environment (soil) at the distant site for cancer cells to seed and colonize. We refer to this phenomenon as chemotherapy-exacerbation of metastasis. Mechanistic studies showed that chemotherapy exerts its pro-cancer effect, at least in part, by modulating macrophages and endothelial cells. In addition, *Atf3*, a stress-inducible gene, in the host cells is an important mediator for chemotherapy to bring about its pro-metastatic effect. Many questions remain. How applicable are these findings to different chemotherapeutic agents and cancers? How will combination chemotherapy using multiple drugs affect the data? Is *Atf3*, a hub of the cellular adaptive-response network, a common element for seemingly different stressors, such as chemotherapy and traumatic injury to enhance metastasis? Should the data from mouse models influence clinical practices? Clearly, much more investigation is required before any clinical practice should be changed. However, current literature suggests that strategies to target the tumor microenvironment, particularly the TAMs, may improve chemotherapy as discussed in previous reviews [5,6,7]. An application of this idea is the use of RG7155, a monoclonal antibody that inhibits CSF-1 receptor activation and thus inhibits macrophage survival and function. In a small study of seven cancer patients, this antibody—used in combination with paclitaxel—was shown to improve the response [71]. One advantage of targeting host cells, rather than cancer cells, is that host cells have stable genomes and are less likely to evade chemotherapy, due to mutations as in the case of cancer cells. However, macrophages constitute a first line of defense for the immune system; thus, targeting them is likely to compromise the immune defense mechanisms. For a review on anti-macrophage therapies, see Reference [72]. Before the idea of targeting tumor microenvironment becomes a clinical reality for cancer treatment, the lessons we learned from mouse models may still be useful. As an example, before the administration of neoadjuvant chemotherapy, the leukocyte complexity in the tumor microenvironment may be a factor to consider (see Section 4.1). As for adjuvant chemotherapy, it is the standard of care for operable diseases. In light of the ability of chemotherapy to promote a favorable tissue environment at the distant sites in mouse models, it is prudent to consider the potential relevance of this finding to human. If we can elucidate the mechanisms behind this observation, we may be able to dampen the undesirable effects of chemotherapy, and thus improve its efficacy.

## Figures and Tables

**Table 1:** Examples of macrophage modulations by chemotherapy to elicit pro-cancer effect.

Macrophage Actions	Some Key Points	References
(a) Alter cancer cell behavior (or activity)	Chemotherapy increases tumor-associated macrophages (TAMs), which protect cancer cells from chemotherapy-induced cell death in a cathepsin-dependent manner.	Shree et al., 2011 [20].
	Chemotherapy increases TAMs in the primary tumors. These TAMs enhance the TIC properties of cancer cells as evidenced by tumorigenic potential, TIC markers, and tumor spheroid formation.	Mitchem et al., 2012 [18]
	Tie2-expressing macrophages (TEMs), a subset of macrophages, isolated from primary tumors of mice treated with chemotherapy stimulated cancer cell invasion in a co-culture assay more efficiently than those isolated from control treated mice.	Chang et al., 2017 [27]
(b) Suppress cytotoxic CD8^+^ T cells	Chemotherapy increases TAMs in the primary tumors. These TAMs suppresses the anti-cancer cytotoxicity of T cells.	Mitchem et al., 2012 [18]
	Chemotherapy increases TAMs in the primary tumors. These TAMs secrete IL10, which reduces the expression of IL12 in dendric cells, leading to the suppression of cytotoxic T cells.	DeNardo et al., 2011 and Ruffell et al., 2014 [17,35]
	Chemotherapy activates the inflammasome in the TAMs, resulting in their secretion of IL1β. IL1β in turn stimulates CD4^+^ T cells to secrete IL17, leading to T cell suppression.	Bruchard et al., 2013 [36]
	Chemotherapy drives the expansion of iM (F4/80, Ly6C+, CCR2+), which suppresses the anti-cancer cytotoxicity of T cells.	Ding et al., 2014 [37]
(c) Alter blood or lymphatic vessels	Chemotherapy induces TEMs (F4/80^+^, Tie2^hi^, CXCR^hi^) to accumulate around the blood vessels, leading to revascularization and tumor growth.	Hughes et al., 2015 [38]
	Chemotherapy increases the abundance of TMEM, which is a site for cancer cells to enter the blood stream. The result is increased circulating cancer cells and metastasis.	Chang et al., 2017 and Karagiannis et al., 2017 [27,28]
	Chemotherapy increases the plasma concentration of VEGF-C, with macrophage as a source of this angiogenic cytokine. Through the VEGF-C/VEGFR3 axis, chemotherapy modulates the lymphatic endothelial cells, leading to increased lymphogenesis and metastasis.	Alishekevitz et al., 2016 [26]

Footnote: In general, more than one chemotherapeutic agent was use in the studies, including paclitaxel, cyclophosphamide, doxorubicin, and gemcitabine.

## Abbreviations

ATF3	Activating transcription factor 3
CD	Cluster of differentiation
iM	Inflammatory monocyte
TAM	Tumor associated macrophage
TEM	Tie2-expressing macrophage
TIC	Tumor initiation cell
TLR	Toll-like receptor
TMEM	Tumor microenvironment metastasis
WT	Wild type
